# Dataset to support the adoption of social media and emerging technologies for students’ continuous engagement

**DOI:** 10.1016/j.dib.2020.105926

**Published:** 2020-06-25

**Authors:** Oluwatobi Noah Akande, Taofeeq Alabi Badmus, Akinyinka Tosin Akindele, Oladiran Tayo Arulogun

**Affiliations:** a Computer Science Department, Landmark University, Kwara State, Nigeria; b School of E-learning Projects, Kampala International University, Kampala, Uganda; c Resilient Engineering Research Group, Faculty of Engineering, University of Nottingham, England, United Kingdom; d Open and Distance Learning Centre, Ladoke Akintola University of Technology, Ogbomoso, Oyo State Nigeria; e Department of Computer Engineering, Federal University of Oye-Ekiti, Ekiti State, Nigeria

**Keywords:** Social media, Emerging technologies, Education technology, Curriculum development, Instructional Design, Online Learning

## Abstract

The recent advancements in ICT have made it possible for teaching and learning to be conducted outside the four walls of a University. Furthermore, the recent COVID-19 pandemic that has crippled educational activities in all nations of the world has further revealed the urgent need for academic institutions to embrace and integrate alternative modes of teaching and learning via social media platforms and emerging technologies into existing teaching tools. This article contains data collected from 850 face to face University students during the COVID-19 pandemic lockdown. An online google form was used to elicit information from the students about their awareness and intention to use these alternative modes of teaching and learning. The questions were structured using the Unified Theory of Acceptance and Use of Technology (UTAUT) model. This data article includes the questionnaire used to retrieve the data, the responses obtained in spreadsheet format, the charts generated from the responses received, the Statistical Package of the Social Sciences (SPSS) file, the descriptive statistics, and reliability analysis computed for all the UTAUT variables. The dataset will enhance understanding of how face to face students use social media platforms and how these platforms could be used to engage the students outside their classroom activities. Also, the dataset exposes how familiar face to face University students are to these emerging teaching and learning technologies. The challenges that could inhibit the adoption of these technologies were also revealed.

**Specifications Table**

**Table:** 

Subject	Education
Specific subject area	Education Technology
Type of data	Text files and SPSS file, instrument, survey data, Charts
How data were acquired	The data in this article was obtained from 850 face to face University students using an online google form.
Data format	Raw data and SPSS data
Parameters for data collection	The data collected is related to students' perceptions and awareness about the use of social media and emerging technologies for teaching and learning. The data includes: how long they have been using a social media account, how often they access their social media account, how familiar they are to social media platforms and emerging technologies like zoom, Microsoft's Team, Facebook Live, Google Classroom, Massive Open Online Courses (MOOC), Augmented (Virtual) Reality & Simulations, etc. What could be the challenges and their perspectives on the impact social media and emerging technologies could have if adopted as a teaching and learning tool.
Description of data collection	The data was collected among face to face University students by administering a questionnaire via an online google form. The questions were structured using the UTAUT model and all the questions were made compulsory for the respondents to avoid missing items.
Data source location	Ogbomoso, Oyo State, Nigeria.
Data accessibility	Data is publicly available at http://dx.doi.org/10.17632/vb2m5×5xhr.3

**Value of the Data**

• The dataset is valuable as it provides insight into how familiar face to face students are to several social media platforms and other emerging technologies that could be used for teaching and learning
• The dataset also helps to understand the challenges students could face if certain social media and emerging technologies are to be adopted for teaching and learning.
• This data will help policymakers for academic institutions to know the best social media platform and emerging technology that could be adopted to enhance teaching and learning as well as to continuously engage students outside the classroom.
• The dataset provides raw data for comparison with other survey data involving the use of social media and emerging technologies by students
• This dataset focused on face to face University students, however, the same raw data can be replicated to capture the opinion of students in other models of education.
• The dataset set illuminates how society has changed with social networks becoming a tool, not only for social interactions but also for continuous learning. Therefore, the data and questionnaire instrument can be valuable in similar studies in the future.

## 1 Data description

**Fig. 1:**
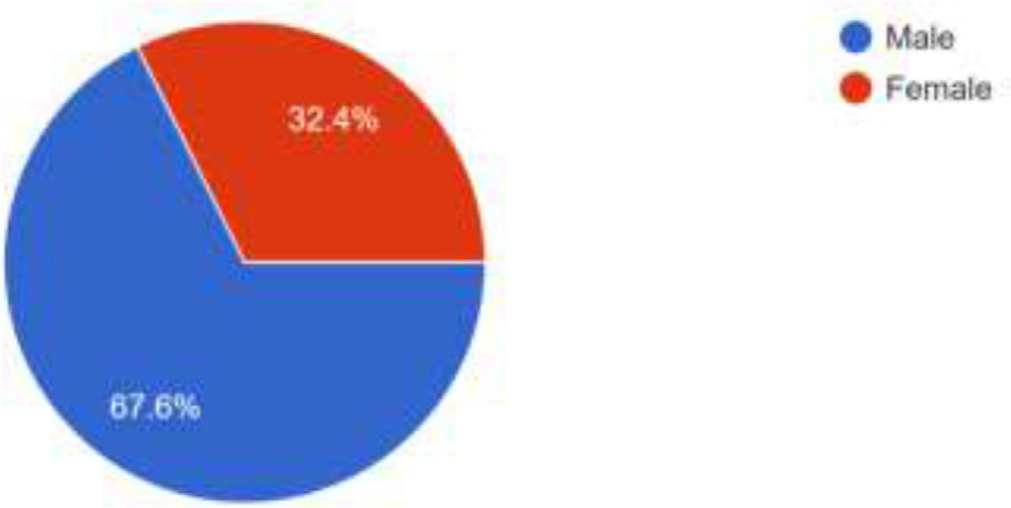
Gender of Respondents.

**Fig. 2:**
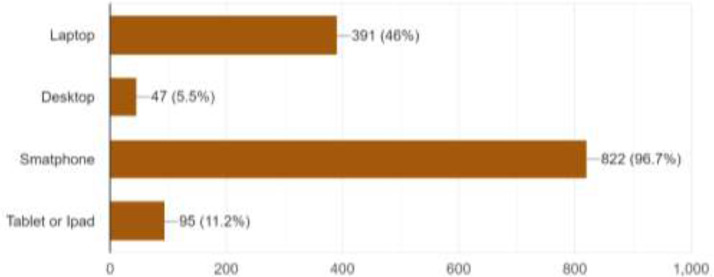
How Respondents Access their Social Media Platforms.

**Fig. 3:**
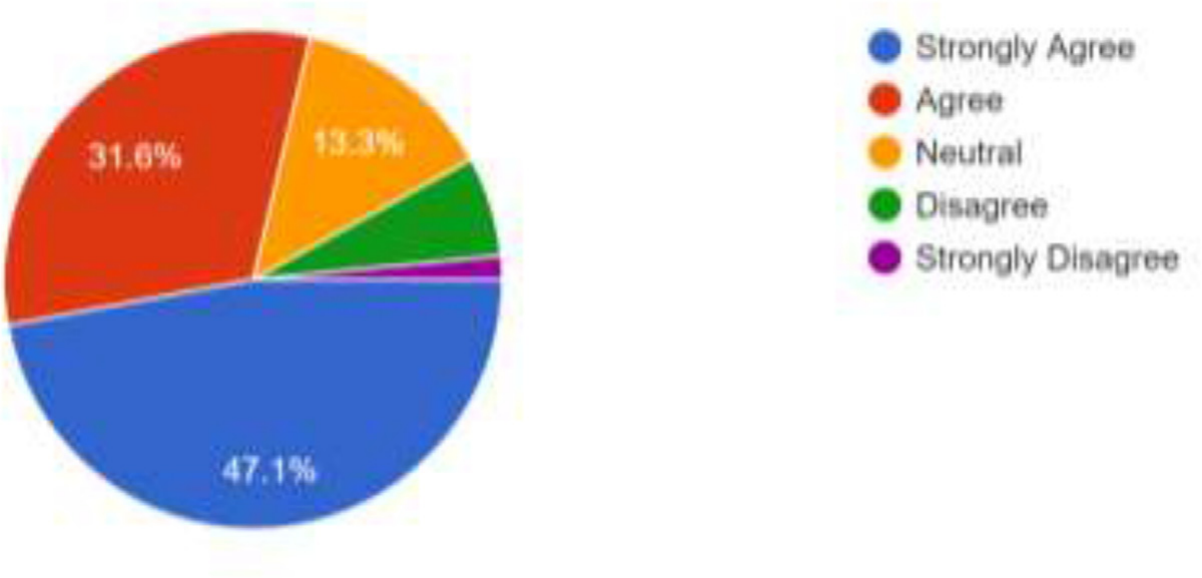
Respondents Response to the Effects of Power Availability.

**Fig. 4:**
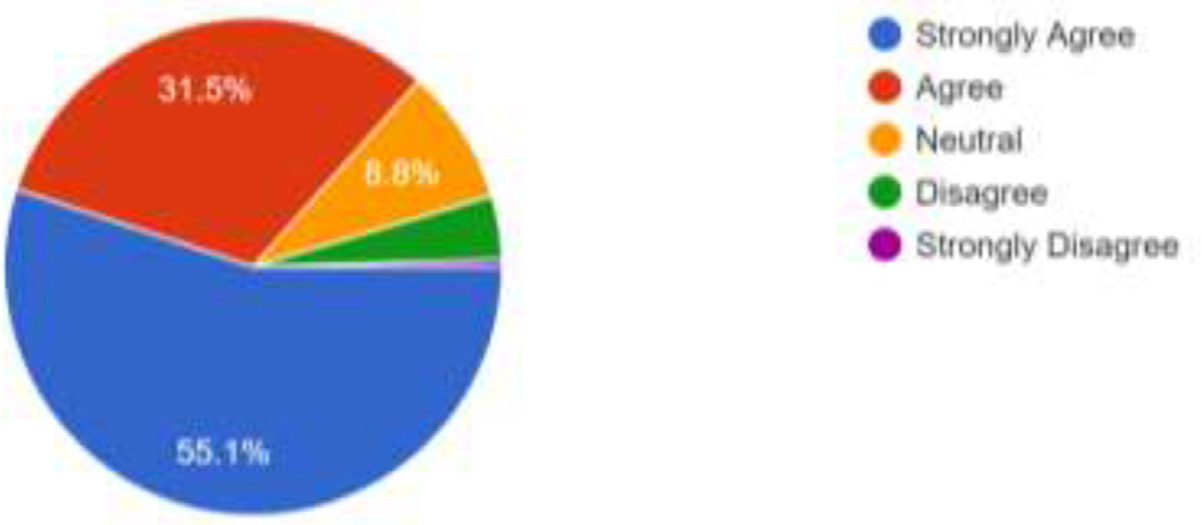
Respondents View on Effects of Internet Data Bundle.

**Fig. 5:**
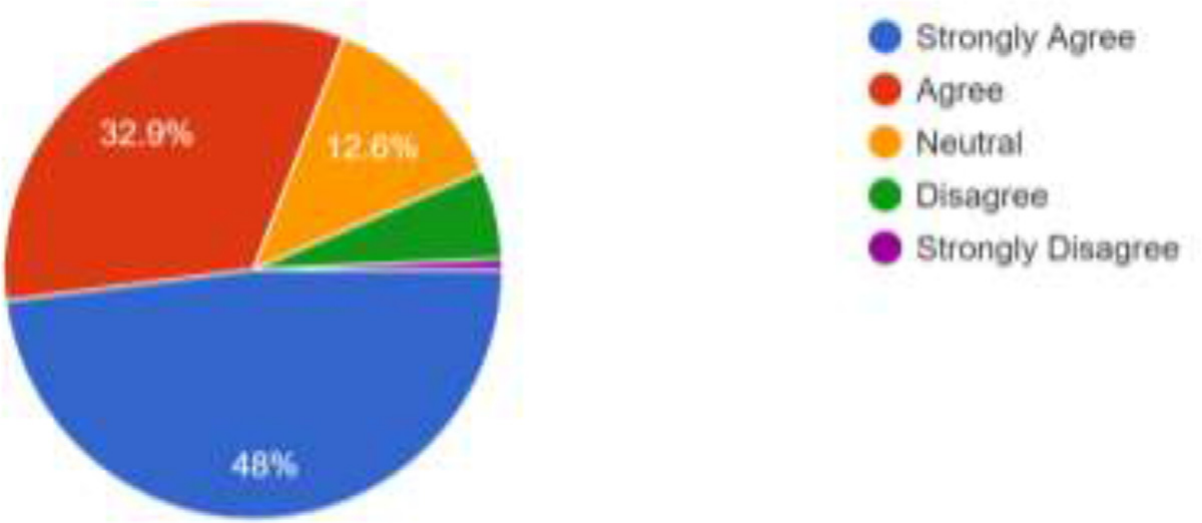
Respondents View on Internet Availability and Signal Strength.

The data included in this article was intended to elicit information about the familiarity of face to face University students to several social media platforms and emerging technologies that could be adopted to facilitate their learning outside the four walls of the classrooms. The online questionnaire was administered among face to face students of Ladoke Akintola University of Technology (LAUTECH), Ogbomoso, Nigeria. It was administered between 19^th^ April and 13^th^ May 2020 when the students were observing COVID-19 lockdown. From the questionnaire data, there are 850 participants with 575 males (67.6%) and 275 females (32.4%) as shown in Fig. 1. A larger percentage of the respondents employ smartphones than laptops in accessing their social media accounts as shown in Fig. 2. This correlates with the thought of Schindler et al., (2017) that smartphones are affordable when compared to laptops, hence, users intend to use them for their internet activities. Also, from the student's perspectives, the data acquired throws more light on the challenges that could limit students from embracing the use of these alternative modes of learning. As shown in Fig. 3, when respondents were asked if power supply could be a major challenge in using social media and emerging technologies for learning, 400 of the respondents (47.1%) strongly agree, 269 (31.6%) agree while 55 respondents (6.5%) disagree. This is certainly due to irregular supply of electricity in most states of the country. Similarly, Fig. 4 reflects that acquisition of internet data bundles could be a challenge in the use of social media and emerging technologies for learning. 468 (55.1%) of the respondents strongly agree to this while 268 (31.55%) respondents agree. Only 4.1% disagree. This is due to the huge cost of data subscription in Nigeria [1]. In addition to availability of data bundles, network signal strength could also be a major challenge especially in remote areas. Therefore, respondents were asked if they think internet availability and signal strength could be a threat to the use of social media platforms and emerging technologies for learning. As shown in Fig. 5, 408 respondents (48%) strongly agree while 280 respondents (32.9%) also agree. Only 49 respondents (5.76%) disagree.

**Fig. 6:**
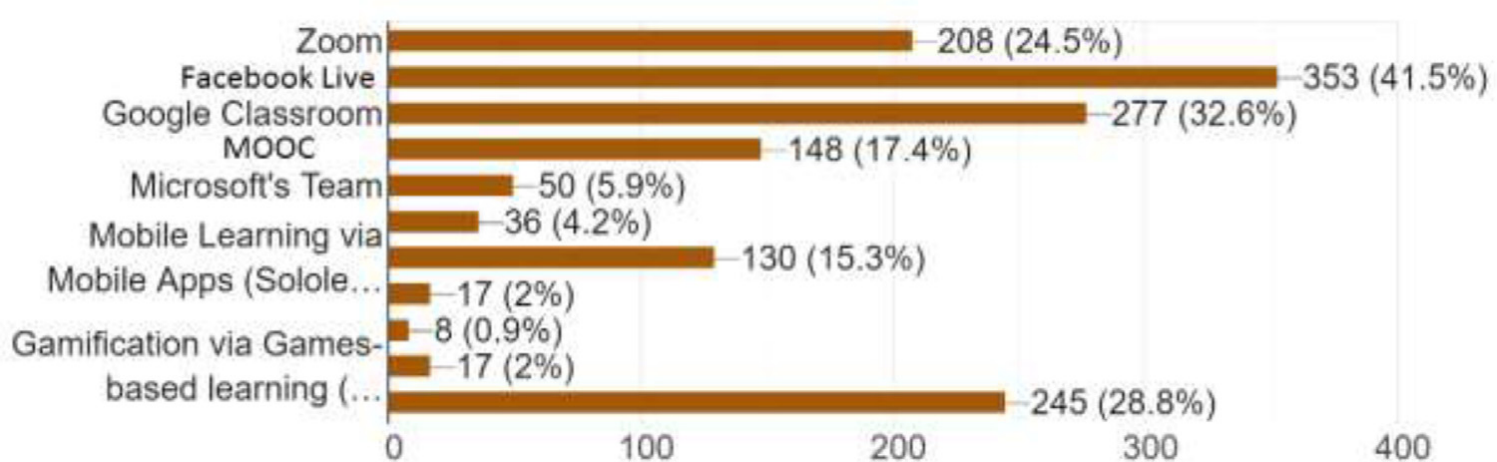
Emerging Technologies Frequently used by Respondents.

**Fig. 7:**
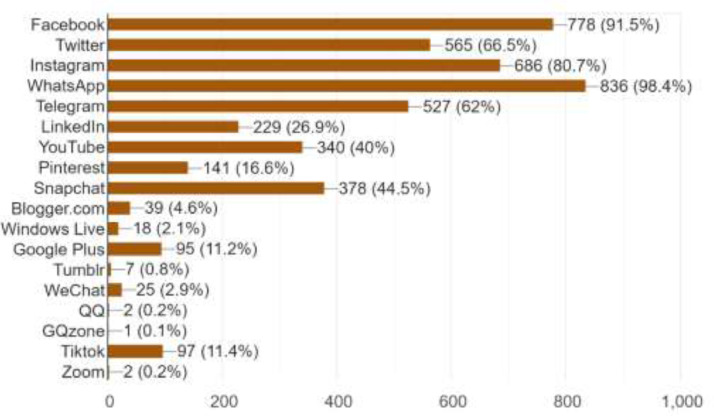
Social Media Platforms Frequently used by Respondents.

Furthermore, the familiarity of the respondents to several social media platforms and emerging technologies were evaluated. Fig. 6 revealed that Facebook Live, Google Classroom, web-based learning platforms, Zoom, and Microsoft's Team are the topmost emerging technologies that respondents are familiar with. This data can help policymakers make informed decisions about the best social media platform and emerging technologies that could be adopted for students’ continuous engagement. Fig. 7 revealed that Facebook, Twitter, Instagram, WhatsApp, Telegram, Snapchat, and Youtube are the topmost social media platforms that respondents are familiar with. Also, responses from other questions structured using the UTAUT Model is provided as supplementary material available in this data article.

## 2 Experimental design, materials and methods

**Fig. 8:**
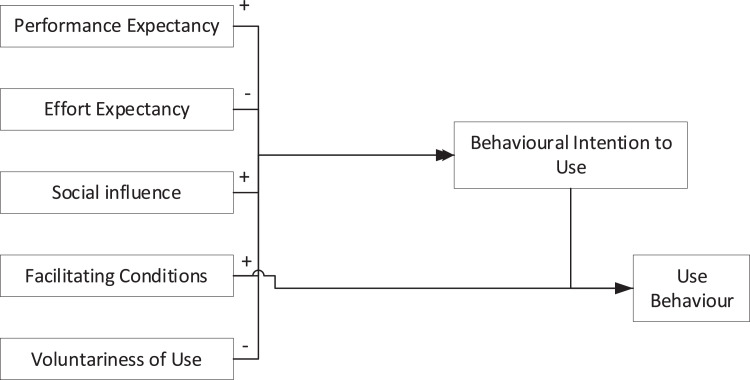
UTAUT model [4].

The study utilized a questionnaire-based survey instrument to retrieve information from Eight hundred and Fifty (850) respondents. The questionnaire was administered via an online google form whose link was sent to student's mail and WhatsApp groups. To ensure there are no missing items, all questions were made compulsory for all respondents to complete. The questions are mainly closed-ended questions in three sections: A, B and C. Section A was used to gather data on five variables describing respondents demographic data such as gender, age, geographic location, academic status and level of education; Eight (8) questions in Section B were tailored at eliciting information about respondents’ awareness of social media and emerging technologies while fifteen (15) questions in section C were structured using Unified Theory of Acceptance and Use of Technology (UTAUT) model shown in Fig. 8. UTAUT model is a widely known theory for examining a user's acceptance and intention to use open data technologies [2]. It also investigates factors influencing the use of Information Technology (IT) while at the same time taking social factors into account [3]. Questions based on the UTAUT model uses metrics such as effort expectancy, performance expectancy, social influence, facilitating conditions, and voluntariness of use. So, fifteen (15) questions in section C were based on these UTAUT metrics. Furthermore, a 5-Point Likert scale starting from 1 for Strongly Disagree and ending with 5 for Strongly Agree was used. Details of these questions are provided in the supplementary documents.

## 3 Statistical significance of the data

To evaluate the statistical significance of the data, descriptive characteristics, and reliability analysis of the fifteen (15) UTAUT variables were computed using SPSS. The descriptive characteristics define the frequency, mean, standard deviation, variance, minimum and maximum, measures of Kurtosis, and skewness of the responses received from each UTAUT variable. The reliability statistics generate the actual value of Cronbach's alpha. This value ranges between 0 and 1. The closer the value is to 1, the higher the internal consistency of the variables. According to [5], a Cronbach's alpha value less than 0.5 is unacceptable, while a value greater than 0.5 is poor, a value greater than 0.6 is questionable while a value greater than 0.7 is acceptable. A value greater than 0.8 is known to be good while a value greater than 0.9 is termed excellent. As provided in Table 1, the Cronbach's Alpha for the 15 UTAUT variables is 0.813 which shows a high level of internal consistency among the variables.

**Table 1:** Reliability Statistics of the UTAUT Variables.

Cronbach's Alpha	Cronbach's Alpha Based on Standardized Items	N of Items
.813	.823	15

Also, the inter-item correlation matrix was computed to establish the internal consistency reliability among the UTAUT variables. It reveals if the variables produce a consistent result. The inter-item correlation value is expected to range between 0.15 and 0.50, values below 0.15 depict that the variables being considered are not correlated while a value greater than 0.5 depicts that the variables are almost a repetition [6]. Finally, item-total statistics of the UTUAT variables were also computed using SPSS. This presents what the scale means, scale variance and Cronbach's alpha will be if a variable is deleted. It also provides the corrected item-total correlation and the squared multiple correlations. Knowing that a high Cronbach's alpha value is preferred, the item-total statistics reveal what happens to Cronbach's alpha if a variable is removed. With this, researchers can know the significant variables and those that can be ignored. The detailed inter-item correlation values and item-total statistics of the UTUAT variables are publicly available on Mendeley via https://data.mendeley.com/datasets/vb2m5x5xhr/3.

## Declaration of Competing Interest

The authors declare that there is no competing interest.

## Appendix Supplementary materials

Image, application 1

Image, application 2
